# Supplementary material for: Missing steps of mitochondrial translation initiation identified in plants
Source: bioRxiv. 2025 Dec 30:2025.12.30.697032. Preprint. [Version 1] doi: 10.64898/2025.12.30.697032 (PMC12776268; doi:10.64898/2025.12.30.697032)
Supplement: Supplement 2 — Supplementary Figure 2: Sequence alignment and structural comparison of (mt)IF3 from Arabidopsis thaliana, Homo sapiens, and Thermus thermophilus. Comparative analysis of mtIF3. a Multiple amino acid sequence alignment of the three (mt)IF3 orthologs generated using Clustal Omega, highlighting conserved residues and main domains. b Superposition of the plant mtIF3/fMet-tRNAiMet complex (mtPIC-3) presented here with H. sapiens mtIF3 (PDB: 6RW4) and T. thermophilus IF3 (PDB: 5LMQ). [file media-2.pdf]

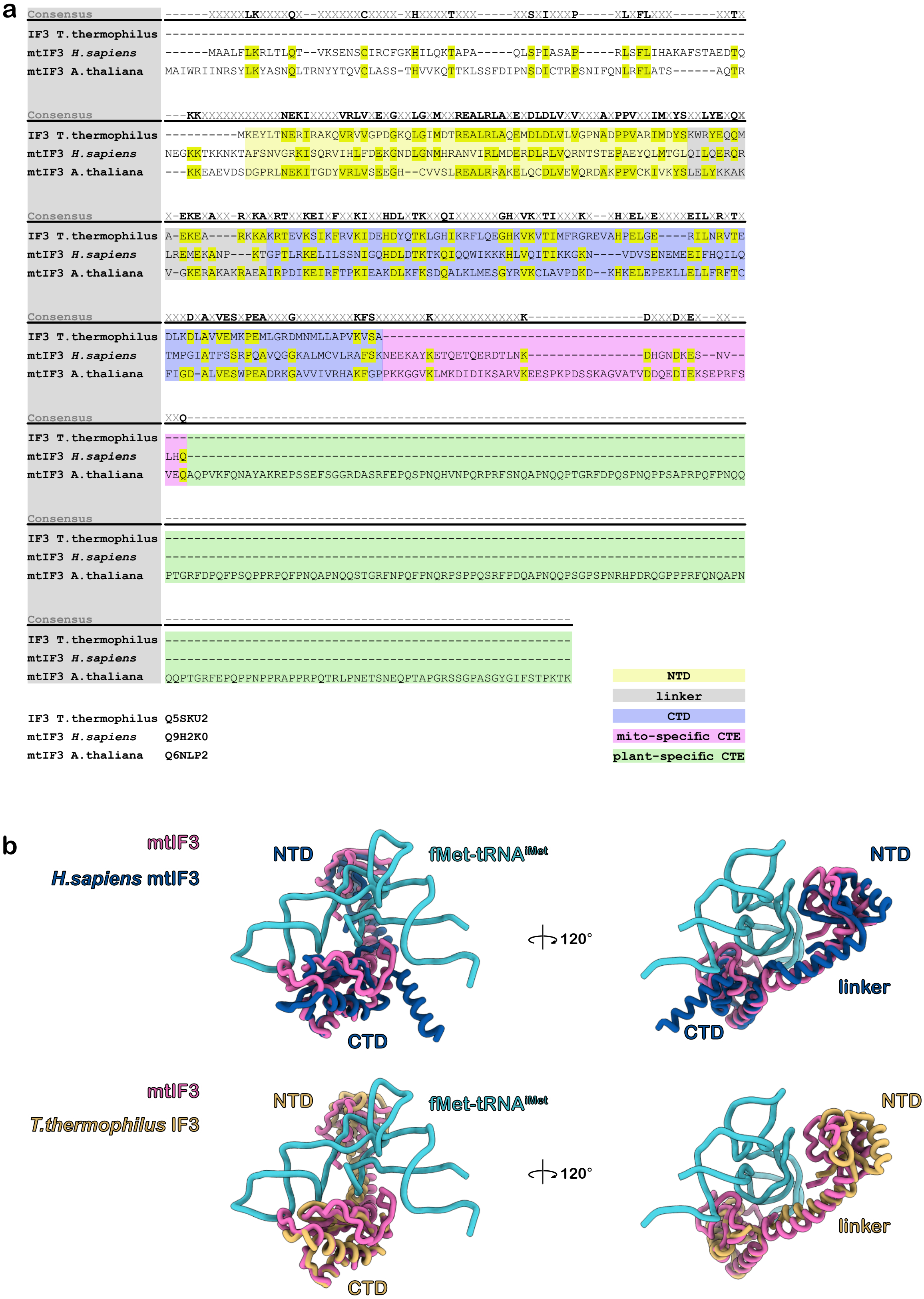

Supplementary Figure 2: Sequence alignment and structural comparison of (mt)IF3 from *Arabidopsis thaliana*, *Homo sapiens*, and *Thermus thermophilus*.

Comparative analysis of mtIF3. **a** Multiple amino acid sequence alignment of the three (mt)IF3 orthologs generated using Clustal Omega, highlighting conserved residues and main domains. **b** Superposition of the plant mtIF3/fMet-tRNA^{iMet} complex (mtPIC-3) presented here with *H. sapiens* mtIF3 (PDB: 6RW4) and *T. thermophilus* IF3 (PDB: 5LMQ).
