# Supplementary material for: Missing steps of mitochondrial translation initiation identified in plants
Source: bioRxiv. 2025 Dec 30:2025.12.30.697032. Preprint. [Version 1] doi: 10.64898/2025.12.30.697032 (PMC12776268; doi:10.64898/2025.12.30.697032)
Supplement: Supplement 5 — Supplementary Figure 5: Single-particle data processing workflow of the mitochondrial pre-initiation complex mtPIC-3. Schematic overview of the data processing workflow. a Pre-processing steps followed by 2D and 3D classification leading to global refinement of mtPIC-3. An orientation distribution plot is shown for the globally refined map. b Local refinements of mtPIC-3, with all masks used indicated. For the final reconstruction, Gold-standard Fourier shell correlation (GSFSC) plots are shown, with resolution determined at the 0.143 threshold. Local resolution maps are displayed on a consistent resolution scale, shown in both front-view and cut-view representations. [file media-5.pdf]

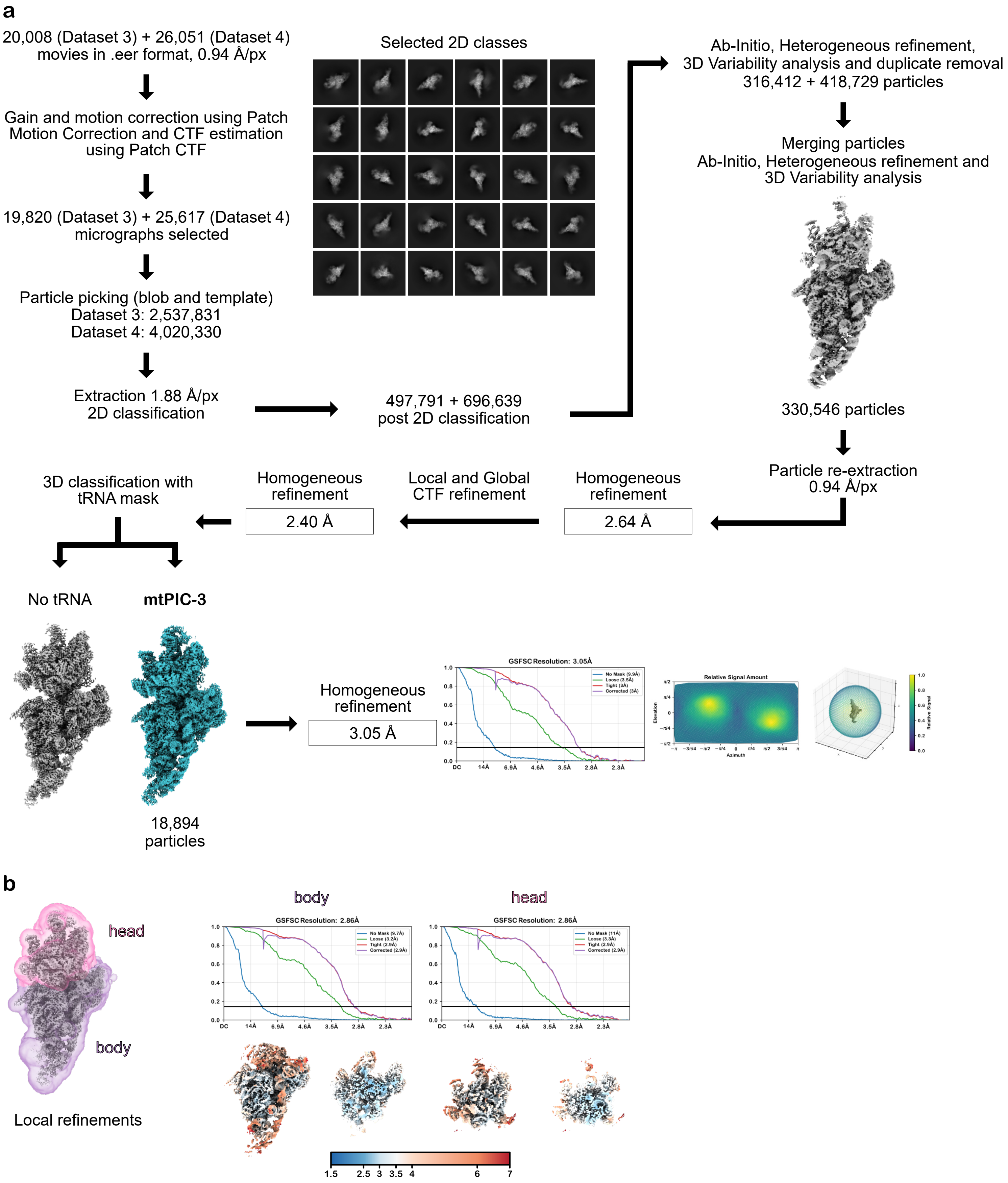

Supplementary Figure 5: Single-particle data processing workflow of the mitochondrial pre-initiation complex mtPIC-3

Schematic overview of the data processing workflow. **a** Pre-processing steps followed by 2D and 3D classification leading to global refinement of mtPIC-3. An orientation distribution plot is shown for the globally refined map. **b** Local refinements of mtPIC-3, with all masks used indicated. For the final reconstruction, Gold-standard Fourier shell correlation (GSFSC) plots are shown, with resolution determined at the 0.143 threshold. Local resolution maps are displayed on a consistent resolution scale, shown in both front-view and cut-view representations.
