# Supplementary material for: Missing steps of mitochondrial translation initiation identified in plants
Source: bioRxiv. 2025 Dec 30:2025.12.30.697032. Preprint. [Version 1] doi: 10.64898/2025.12.30.697032 (PMC12776268; doi:10.64898/2025.12.30.697032)
Supplement: Supplement 7 — Supplementary Table 1: Cryo-EM data collection, refinement and validation statistics. [file media-7.pdf]

	mtPIC-1	mtPIC-2	mtPIC-3	mtIC-1*
**PDB**	**XXXX**	**XXXX**	**XXXX**	**XXXX**
**Unfocused**	**XXXX**	**XXXX**	**XXXX**	**XXXX**
**Data collection and processing**				
Magnification	130,000x	130,000x	130,000x	130,000x
Voltage (kV)	300	300	300	300
Electron exposure ($e^-/Å^2$)	40	40	40	40
Defocus range (μm)	-0.4 to -2.2	-0.4 to -2.2	-0.4 to -2.2	-0.4 to -2.2
Pixel size (Å)	0.94	0.94	0.94	0.94
Symmetry imposed	C1	C1	C1	C1
Initial particle images (no.)	4,340,523	4,340,523	6,558,161	4,371,444
Final particle images (no.)	141, 355	62, 215	18, 894	18, 503
Map resolution (Å) FSC 0.143	2.28	2.55	3.05	2.92
Map resolution range (Å)	2.2 - 7.3*	2.1 - 8.2*	2.1 - 10.6*	2.1 - 9.3*
**Refinement**				
Initial model used (PDB code)	9GYT	9GYT	9GYT	9EVS
Model resolution (Å) FSC 0.143	2.3	2.55	3.05	2.92
CC Model vs Data (mask)	0.78	0.78	0.79	0.78
Map sharpening *B* factor ($Å^2$)	-30.8	-28.4	-12.9	-22.2
Model composition				
Non-hydrogen atoms	89548	89124	92977	207751
Residues : Protein - Nucleotide	6707 - 1610	6632 - 1619	6863 - 1681	13343 - 4686
Ligands	ATP: 1	ATP: 1	ATP: 1	ATP: 1
B factors ($Å^2$)				
Protein	0.00/235.67/47.95	0.00/235.67/48.35	0.00/540.51/75.72	0.00/444.13/54.33
Nucleotide	1.55/271.67/54.66	1.55/272.81/54.72	8.32/562.91/71.13	0.37/376.89/66.33
Ligand	12.07/76.37/30.71	12.07/76.37/30.71	3.00/95.27/31.17	4.98/97.19/34.70
R.m.s. deviations				
Bond length ($Å^2$)	0.007	0.007	0.007	0.004
Bond angles (°)	1.000	0.998	0.945	0.811
Validation				
MolProbity score	2.06	2.09	2.24	1.90
Clash score	11.86	12.07	16.19	6.46
Poor rotamers (%)	2.20	2.15	2.23	2.71
Ramachandran plot				
Favored (%)	96.64	96.40	96.07	96.60
Allowed (%)	3.18	3.41	3.74	3.34
Disallowed (%)	0.18	0.18	0.19	0.06

* min - 75th percentile, in cryoSPARC Local resolution Estimation

Extended Data Table 1: Cryo-EM data collection, refinement and validation statistics
